# Household Clustering of SARS-CoV-2 in Community Settings: A Study from Rural Ecuador

**DOI:** 10.4269/ajtmh.20-0688

**Published:** 2020-08-04

**Authors:** Oscar H. Del Brutto, Aldo F. Costa, Robertino M. Mera, Bettsy Y. Recalde, Javier A. Bustos, Héctor H. García

**Affiliations:** 1 School of Medicine, Universidad Espíritu Santo, Guayaquil, Ecuador;; 2 Community Center, The Atahualpa Project, Atahualpa, Ecuador;; 3 Department of Epidemiology, Gilead Sciences, Inc., Foster City, California;; 4 Department of Microbiology, Center for Global Health, Universidad Peruana Cayetano Heredia, Lima, Perú

## Abstract

The SARS-CoV-2 pandemic is now expanding into the developing world with devastating consequences. Departing from a population-based study in rural Ecuador where all adult individuals (aged 40 years or older) were tested for SARS-CoV-2 IgG and IgM antibodies, we expanded it to include a house-based case–control component assessing in-house clustering and other variables potentially associated with infection. We selected houses where exactly two study participants lived and were both seropositive (case-houses), and matched 1:1 to control-houses where both were seronegative. Younger household members had an antibody test performed. Infected household members were found in 33 (92%) case-houses and in only six (17%) control-houses. In 28/29 discordant house pairs, the case-house had seropositive household members and the control-house did not (odds ratio: 28; 95% CI: 4.6–1,144). Our data demonstrate strong in-house clustering of infection in community settings, stressing the importance of early case ascertainment and isolation for SARS-CoV-2 control.

The SARS-CoV-2 novel pandemic has now reached most of the developing world.^1–3^ Underdevelopment, with all its consequences, will be likely associated with mass spread of this infection in populations inhabiting rural areas of these countries.^4–6^ Despite abundant information on the mechanisms of transmission of COVID-19 disease, evidence on in-house clustering of SARS-CoV-2 infection is limited to skilled nurse facilities and shelters in developed countries, and to isolated reports of families of index symptomatic cases.^7–9^ There are certainly no studies assessing in-house clustering in community settings.

We recently conducted a population-based study to determine the prevalence of IgM and IgG antibodies against SARS-CoV-2, as well as COVID-19–related clinical manifestations, in 673 community-dwelling middle-aged and older adults enrolled in the Atahualpa Project cohort.^10^ In that study, we demonstrated a seroprevalence of 45% and clinical manifestations consistent with COVID-19 in 77% of the seropositive individuals. Seropositive individuals were disseminated across the entire village, and we noticed a significant association between seropositivity and the use of open latrines, suggesting a contributing role for fecal–oral transmission of the virus.^10^ However, younger individuals were not included in the population-based study, limiting our capacity to evaluate the presence of in-house clustering of infection.

This house-based case–control study expands the aforementioned cohort to include younger members of a sample of households to allow assessment of in-house clustering of seropositive individuals and other variables at the house level. The Independent Review Board of Universidad Espiritu Santo (Institutional Review Board Organization: 0010320; Federal Wide Assurance: 00028878) approved the study protocol and the informed consent forms (signed by parents or guardians in the case of children).

Atahualpa is a rural village located in coastal Ecuador, where several epidemiological studies have been conducted. Characteristics of the population have been detailed elsewhere.^11^ In brief, Atahualpa residents are homogeneous regarding race/ethnicity (Amerindian ancestry), socioeconomic status, and lifestyle. The village has electricity, and almost all houses have piped water, but most streets are not paved. A sizable proportion of houses use open latrines (often located at the backyards) for feces disposal. There is only one health center of the Minister of Health (ambulatory medical care), and the nearest hospital is about 10 miles away in a small city (Ancón).

For this case–control study, houses where two participants of the Atahualpa Project live and both were seropositive (case-houses) were paired 1:1 with neighboring houses where two participants of the Atahualpa Project live, and both were seronegative (control-houses). Selected houses were evenly distributed across the village (Figure 1). Another requisite for considering a house as a case or a control was the presence of two (±1) additional individuals aged < 40 years, living in the same house. The number of two adult participants and one to three younger individuals per house was selected to make the sample of this case–control study homogeneous. The outcome of this study was defined as the number of households with at least one seropositive young individual (aged < 40 years), compared between houses with two seropositive adults versus houses with two seronegative adults.

**Figure 1.**
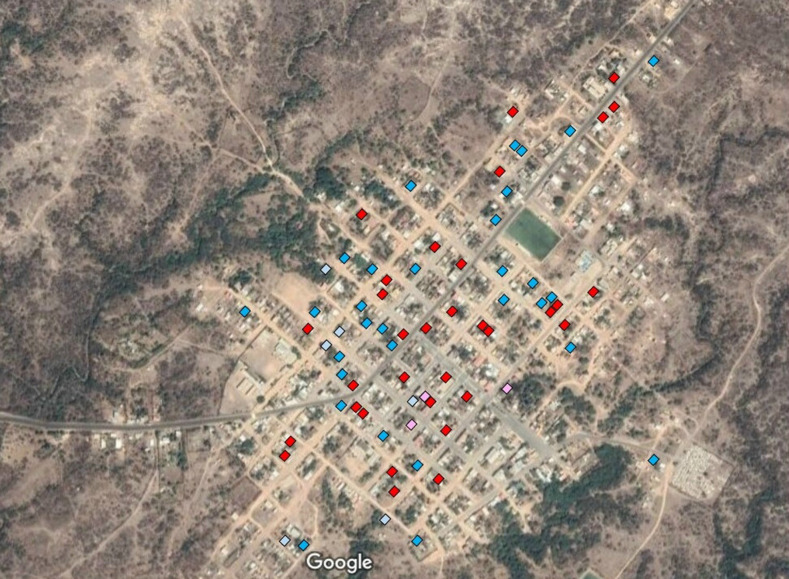
Google map of Atahualpa (Google Earth, Google Inc., Mountain View, CA) showing the distribution of selected houses. Positive case-houses are marked in red, negative case-houses in pink, negative control-houses in blue, and positive control-houses in light blue.

Detection of SARS-CoV-2 IgM and IgG antibodies was performed by the use of BIOHIT SARS-CoV-2 antibody test kit, colloidal gold method (BIOHIT HealthCare Ltd., Cheshire, United Kingdom). The results of those tests were independently reviewed by two readers of our group, with excellent (> 0.90) Kappa coefficients for inter-rater agreement. Discrepancies were resolved by consensus with the help of a third reviewer.

Because in this case–control study the house was the unit of analysis, we selected covariates that may influence household transmission of the disease across middle-aged and older adults and their younger counterparts. Covariates investigated were the number of bedrooms per house, the use of open latrines (instead of having a flushing toilet system), and the presence of disparities according to the household components of the social determinants of health (economic status and housing).^12^

The presence of at least one seropositive younger adult (positive outcome) across case- and control-houses was compared by the use of the McNemar’s test for correlated proportions (matched-pair analysis). A conditional logistic regression model, adjusted for the aforementioned covariates, was fitted for multivariate analyses.

Figure 2 is a flowchart depicting the process of house selection and the reasons for not including potentially eligible houses. Of 411 houses identified in the aforementioned population-based study, 74 (18%) met the inclusion criteria for this study. Of these, 38 were case-houses, and the remaining 36 were control-houses. Two of the 38 case-houses were randomly excluded using the final three digits of a random numbers list, leaving 72 houses for matching pair analysis (36 case-houses and 36 control-houses). Each pair consisted of a case-house and the nearest located control-house (often in the same block).

**Figure 2.**
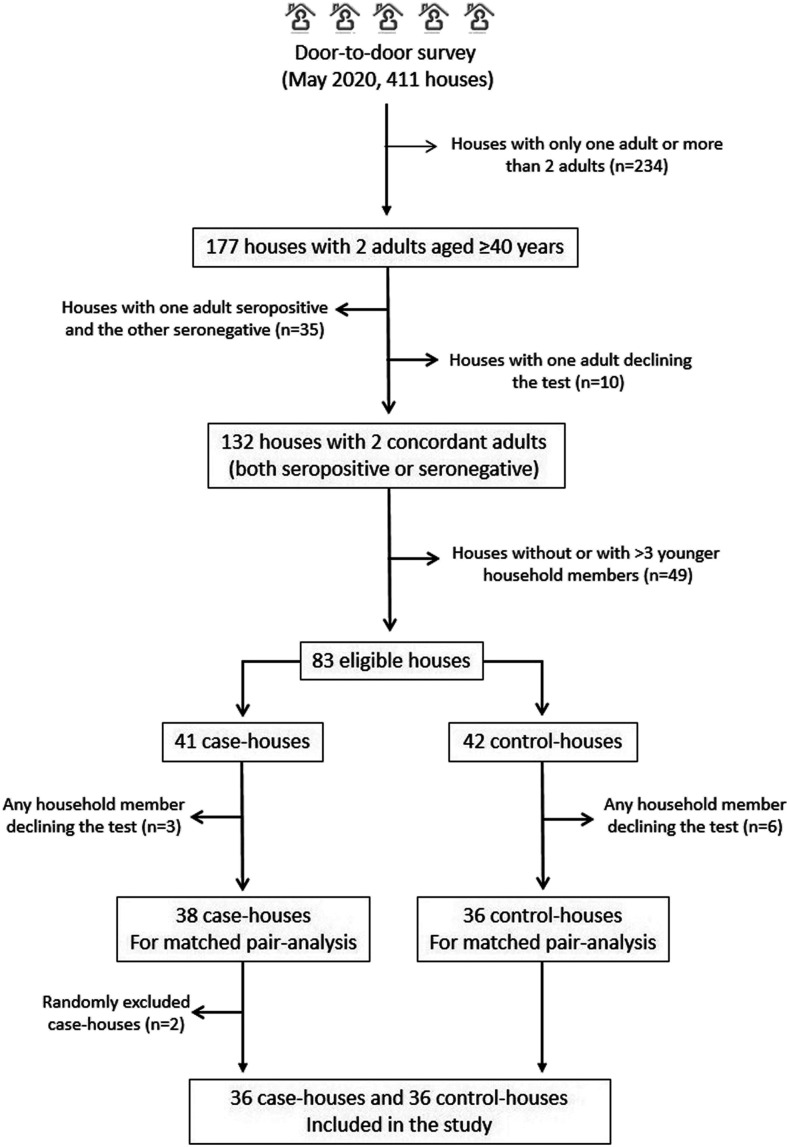
Flowchart depicting the process of house selection and the reasons for not including potentially eligible houses.

As per the protocol, both case-houses and control-houses had two middle-aged and older adults per house (144 individuals). The mean (±SD) age of middle-aged and older adults living in case-houses was 60.7 ± 10.8 years and that of those in control-houses was 57.4 ± 9.8 years with only a marginal difference across groups (*P* = 0.057). There were no differences in the percentage of women living in case-houses or control-houses (53% versus 51%, *P* = 0.867).

A total of 145 individuals younger than 40 years were included in this study (74 in case-houses and 71 in control-houses), with a mean (±SD) of 2 ± 0.8 per house. Twenty-five of the houses had only one, 21 had two, and the remaining 26 had three young individuals, with no differences in the mean number of young individuals across case-houses and control-houses (2.06 ± 0.81 versus 1.97 ± 0.87, *P* = 0.520).

The mean (±SD) age of the 74 younger individuals living in case-houses was 21.1 ± 11.5 years and that of the 71 younger individuals living in control-houses was 18.7 ± 9.4 years (*P* = 0.172). There were also no differences in the percentage of young women living in case-houses versus control-houses (54% versus 59%, *P* = 0.536). Fifty-four (73%) of 74 younger household members in case-houses were seropositive, as opposed to only nine (13%) of 71 younger household members in control-houses (*P* < 0.001). In cases with more than one individual aged < 40 years, it was common to observe that all of them were seropositive in case-houses and seronegative in control-houses (Figure 3). There was at least one individual younger than 40 years infected in 33 (92%) case-houses compared with six (17%) control-houses (*P* < 0.001).

**Figure 3.**
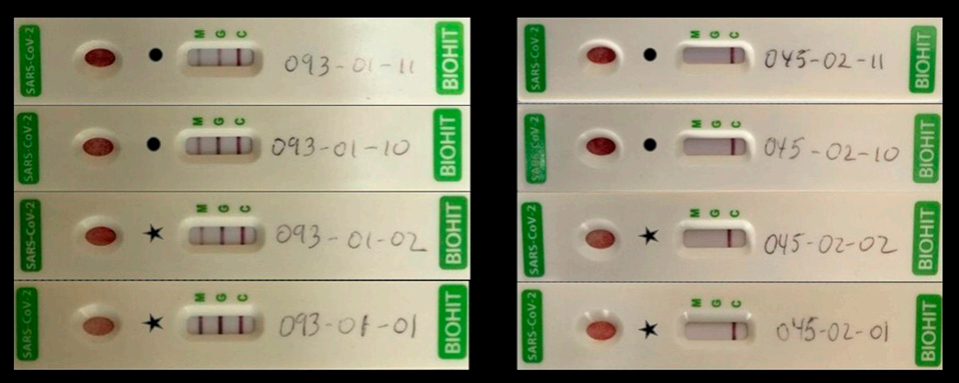
Serological results of household members of a case-house (left panel) and a control-house (right panel). When both adult members were seropositive (black dots), it was common to observe that younger members were all positive (black stars). The opposite was often seen when adults were both seronegative.

Matched-pair data disclosed seven concordant pairs (five pairs in which both the case- and the control-houses were positive, and two pairs in which neither was positive), as well as 29 discordant pairs; in 28 (97%) of these pairs, the case-house was positive, but the control-house did not. The odds ratio (OR) for having at least one seropositive individual younger than 40 years in case-houses compared with control-houses was 28 (95% CI: 4.6–1,144, *P* < 0.001, McNemar’s test for correlated proportions).

A conditional (fixed-effects) logistic regression model, adjusted for the aforementioned covariates, demonstrated that the association between having seropositive adults and younger individuals in the same house was highly significant (OR: 49.1; 95% CI: 3.1–784, *P* = 0.006). None of the included covariates remained independently significant in this model (Table 1). An additional conditional logistic regression model, including the number of household members younger than 40 years as a covariate, further increased the OR estimate for case-houses away from no effect.

**Table 1:** Conditional (fixed-effects) logistic regression model demonstrating an independent significant association between having seropositive adults and seropositive younger individuals in the same house

	Odds ratio (95% CI)	*P*-value
At least one seropositive individual aged < 40 years (outcome)	49.14 (3.08–784)	0.006
Number of bedrooms	0.74 (0.17–3.16)	0.683
Having a flushing toilet system	0.19 (0.17–3.16)	0.212
Income lower than minimum wage	1.87 (0.18–19.88)	0.604
Poor housing	1.38 (0.11–16.85)	0.800

The magnitude and consequences of the SARS-CoV-2 pandemic in remote rural settings are largely unknown. Indeed, two investigators from our group independently conducted a comprehensive literature search for publications on community-based epidemiological studies of SARS-CoV-2 in developing countries, using relevant key words, identifying no high-quality published or preprinted population-based study assessing the prevalence of infection by SARS-CoV-2 in community-based settings in developing countries. We only found four preprints on serological surveys, one from Iran, one from South Africa, and two from Brazil, all taking probabilistic samples in small fractions of the studied populations.^13–16^ The design of those studies does not allow to ascertain the presence of in-house clustering.

The present study demonstrates strong in-house clustering of seropositive cases in a rural community, as are much of the areas now being hit by the pandemic. The magnitude of this in-house clustering stresses the importance of involving early case ascertainment and isolation in SARS-CoV-2 control policies, particularly in developing countries. This study also supports previous fears of mass spread of the disease in rural populations of the developing world, which seem to be not prepared for this pandemic at all.^17–19^
